# In vivo imaging of epithelial wound healing in the cnidarian *Clytia hemisphaerica* demonstrates early evolution of purse string and cell crawling closure mechanisms

**DOI:** 10.1186/s12861-017-0160-2

**Published:** 2017-12-19

**Authors:** Zach Kamran, Katie Zellner, Harry Kyriazes, Christine M. Kraus, Jean-Baptiste Reynier, Jocelyn E. Malamy

**Affiliations:** 1 Biological Sciences Collegiate Division, The University of Chicago, 924 East 57th Street, Chicago, IL 60637 USA; 2 Niles North High School, District 219, 7700 Gross Point Rd., Skokie, IL 60077 USA; 3 Department of Molecular Genetics and Cell Biology, The University of Chicago, 929 East 57th Street, Chicago, IL 60637 USA

**Keywords:** Cnidaria, Epithelial wound healing, Basement membrane, Purse string, Cell crawling, Clytia

## Abstract

**Background:**

All animals have mechanisms for healing damage to the epithelial sheets that cover the body and line internal cavities. Epithelial wounds heal either by cells crawling over the wound gap, by contraction of a super-cellular actin cable (“purse string”) that surrounds the wound, or some combination of the two mechanisms. Both cell crawling and purse string closure of epithelial wounds are widely observed across vertebrates and invertebrates, suggesting early evolution of these mechanisms. Cnidarians evolved ~600 million years ago and are considered a sister group to the Bilateria. They have been much studied for their tremendous regenerative potential, but epithelial wound healing has not been characterized in detail. Conserved elements of wound healing in bilaterians and cnidarians would suggest an evolutionary origin in a common ancestor. Here we test this idea by characterizing epithelial wound healing in live medusae of *Clytia hemisphaerica*.

**Results:**

We identified cell crawling and purse string-mediated mechanisms of healing in *Clytia* epithelium that appear highly analogous of those seen in higher animals, suggesting that these mechanisms may have emerged in a common ancestor. Interestingly, we found that epithelial wound healing in *Clytia* is 75 to >600 times faster than in cultured cells or embryos of other animals previously studied, suggesting that *Clytia* may provide valuable clues about optimized healing efficiency. Finally, in *Clytia*, we show that damage to the basement membrane in a wound gap causes a rapid shift between the cell crawling and purse string mechanisms for wound closure. This is consistent with work in other systems showing that cells marginal to a wound choose between a super-cellular actin cable or lamellipodia formation to close wounds, and suggests a mechanism underlying this decision.

**Conclusions:**

1. Cell crawling and purse string mechanisms of epithelial wound healing likely evolved before the divergence of Cnidaria from the bilaterian lineage ~ 600mya 2. In Clytia, the choice between cell crawling and purse string mechanisms of wound healing depends on interactions between the epithelial cells and the basement membrane.

**Electronic supplementary material:**

The online version of this article (10.1186/s12861-017-0160-2) contains supplementary material, which is available to authorized users.

## Background

The epithelium is made up of one or more layers of epithelial cells covering the external surface of the body and lining internal cavities. Epithelial cells in organs such as the skin, the intestine and the cornea form sheets of closely associated cells. The epithelial cell sheet(s) can be damaged by environmental injury (cuts, burns, scrapes), surgery, and by normal events such as food absorption in the case of intestinal epithelium [1, 2]. In all cases, gaps must be rapidly closed to prevent infection and disease [3]. Therefore, understanding how cells in an epithelial sheet recognize and rapidly close a wound is of tremendous importance, both as a fundamental biological question and as a factor in human health.

Much of our understanding of epithelial wound healing comes from monolayers of epithelial cells grown in tissue culture, which can be imaged at high resolution. In a now classic in vitro assay, cell monolayers are manually manipulated (scratched, poked, ablated with a laser) to simulate wounding. More recently, researchers developed stencils to create gaps in sheets of undamaged epithelial cells as they grow over a culture substrate, defining a “wound” of specific geometry when the stencil is removed [4]. Wound healing assays in embryos and oocytes (e.g. *Drosophilia*, mouse, *Xenopus*) provide in vivo models where cells can be visualized [5–10] and is of particular interest because the healing rates are so much more rapid than in adult tissues. Corneal epithelial wound healing was an early in vivo model for adult animals (i.e. [11]) and several additional in vivo adult models have recently arisen (e.g. zebrafish and mouse cutaneous wounds [12, 13]). Morphogenic movements in embryogenesis such as *Drosophilia* dorsal closure and *C. elegans* ventral enclosure have also been used as models for the wound healing process [7, 10, 14].

From studies in all these systems, the consensus has emerged that there are two distinct sequences of events that can close epithelial gaps 1) Lamellipodia-dependent cell crawling - Lamellipodia are produced by cells adjacent to the wound (marginal cells) and these cells migrate into the gap; in large wounds, cells behind the marginal cells migrate as well; 2) “Purse string” closure- Actin filaments associated with myosin II form a super-cellular cable around the wound circumference, presumably linking cells through adherens junctions. This cable contracts, drawing the edges of marginal cells inwards [1, 2, 8, 11, 14–19] . In either mechanism, the edges of the cells eventually meet across the shrinking gap and establish junctions to restore the integrity of the epithelial cell sheet. How a choice is made between lamellipodia-dependent cell crawling or purse string mechanisms of wound closure remains a major question in the field. Various studies have suggested that the decision may be affected by wound size, geometry and/or tissue type, with cell crawling reported to dominate in large wounds and wounds with a convex curvature, and purse strings to dominate in small wounds, wounds where there is a concave curvature, and in embryonic tissues (i.e. [7, 20, 21]). However, there are many examples where both cell crawling and purse strings are involved in closure of a wound, and overall the contribution of each to healing remains unclear [1].

While purse string and lamellipodia-dependent cell crawling mechanisms have been demonstrated in vertebrates and invertebrates, it is not known when these mechanisms first evolved. Cnidarians are very simple animals that represent one of earliest extant life forms to diverge from the bilaterian lineage (~600 mya). The small hydrozoan *Clytia hemisphaerica* has been recently adopted as a model for development and molecular evolution, and simple protocols for maintaining the animal in the lab are available [22]. To investigate the evolutionary origin of wound healing mechanisms, we developed an in vivo epithelial wound healing model in *Clytia*, taking advantage of the transparency of the organism and the easy visualization of the large squamous epithelial cells.

Here we show healing of epithelial wounds in *Clytia* by cell crawling and purse string closure, indicating that these processes likely arose in a common ancestor of the Cnidaria and Bilateria. Interestingly, healing is extremely rapid in *Clytia*, exceeding the rate of healing in other model systems by ~75 to >600 fold. Another unique feature of *Clytia* is that a myosin II-dependent contraction of a super-cellular actin cable occurs as a key step in both cell crawling and purse-string wound closure mechanisms. Hence, lamellipodia formation and not contraction of an actin cable distinguishes between the two mechanisms. Finally, the simple structure and ease of imaging in *Clytia* allowed us to observe epithelial wound healing in the presence and absence of the basement membrane. We found that the choice of wound healing mechanism in *Clytia* was not predicted by size or shape but was dependent on the integrity of the basement membrane. When lamellipodia encountered defects in the basement membrane there was a rapid transition from cell crawling to purse-string closure of the wound. This is consistent with in vitro studies in other systems showing that the extracellular matrix plays a role in determining the wound closure mechanism [18, 23], but is the first time to our knowledge that a mechanistic switch from cell crawling to actin purse string in response to basement membrane integrity has been documented in a live animal.

## Results

### Development of a wound healing assay in the cnidarian *Clytia hemisphaerica*

**Fig. 1:**
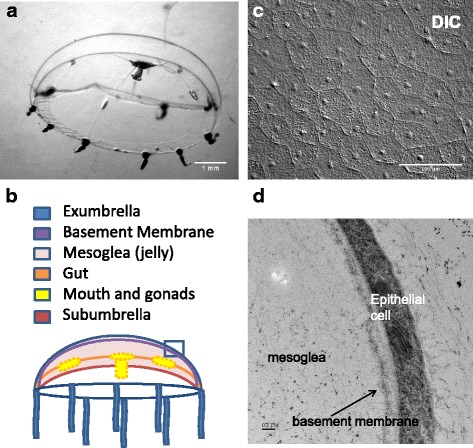
The medusa of *Clytia hemisphaerica* (**a**) ~3 week old animal Scale bar = 1 mm (**b**) Schematic depiction of *Clytia* medusa. The basement membrane underlying the exumbrella epithelial cells is represented by a purple line - basement membranes associated with subumbrella epithelia and endodermal epithelial cells are not shown. **c** DIC image of the surface of the exumbrella in a live medusa Scale bar = 100 μm. **d** Transmission electron micrograph of a section through the exumbrella epithelial sheet in a 1 day old animal, showing part of a cell and the underlying basement membrane and mesoglea. A layer of material covering the surface of the medusa can also be seen. Scale bar = 0.2 μm

Hydrozoans exist primarily in the form of a sessile colony of hydroids or polyps that adhere to rocks, shells, or other structures. Some hydrozoans such as *Clytia hemisphaerica* (*Clytia*) also have a medusa (jellyfish) stage; these medusae swim or drift away from the original colony, releasing gametes at a distance to generate new polyp colonies. In *Clytia*, medusae are released continuously from specialized polyps [22]. Polyp colonies are easily maintained in the lab, and medusae can be collected daily and raised for experiments. The newly released medusae are approx. 0.1 cm in diameter. At 2–3 weeks old, the medusa is ~0.5 cm in diameter, an ideal size for manipulation and imaging (Fig. 1a).

*Clytia* medusae have a monolayer of squamous ectodermal epithelial cells on the exumbrella (upper surface of the bell) and subumbrella (lower surface) (Fig. 1a-c). Beneath the epithelial cells is the mesoglea (jelly), which is ECM-like in composition [24, 25] and makes up the majority of the medusa body. We focused on the non-contractile epithelial cells of the exumbrella. Importantly, these cells and the underlying mesoglea are transparent. Using DIC microscopy, the outlines and nuclei of the squamous epithelial cells are clearly visualized (Fig. 1c). The cells are roughly hexagonal and approximately 50 μm in diameter. Immediately below the exumbrella epithelial cells is a basement membrane (Fig. 1b,d), a specialized region of the extracellular matrix (ECM) that forms a surface and separates the epithelial cells from the rest of the matrix [25, 26].

**Fig. 2:**
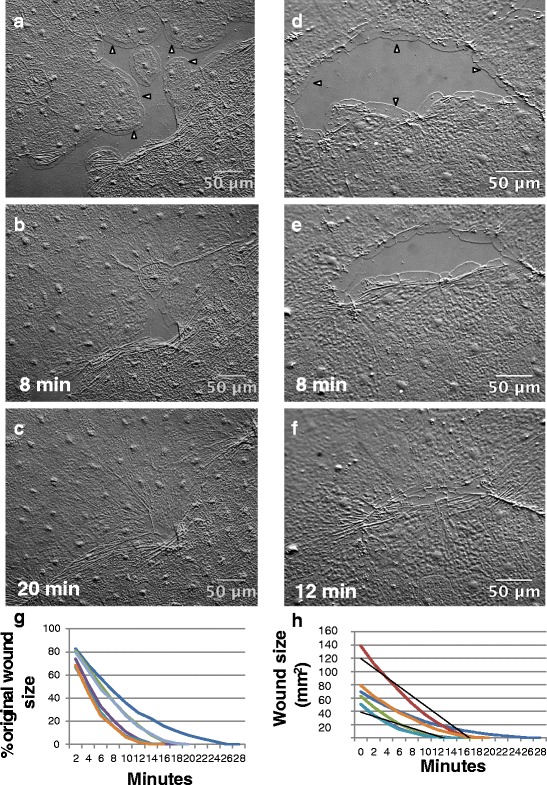
Epithelial wounds in the Clytia medusa exumbrella heal rapidly. Two wounds were imaged at an arbitrary start time (**a**, **d**) and at the indicated times afterwards (**b**-**c**; **e**-**f**). Examples of lamellipodia are indicated with arrowheads (**a**, **d**). Experiment was replicated >10 times. **g**, **h** Closure of 6 independent wounds 50–150 mm^2^ in area are presented as % initial area (**g**) or total area (**h**) over time. Note that the initial area may be less than the original size of the wound, as some time inevitably elapses between wounding the animal and imaging. Area measurements were taken every 2 min. Black lines in (**h**) are fitted to the steepest (red) and shallowest (aqua) curves to approximate the rate of change in wound area. Scale bar = 50 μm

To create wounds in the epithelium of the exumbrella, 2–3 week old *Clytia* medusae were placed on depression slides with the exumbrella facing upwards. We found that manipulation of the animals often caused the epithelial cells to separate, creating wounds. To create larger wounds, we used a drawn Pasteur pipette to gently abrade the surface of the animal. The orientation of the wounds was not controlled, and was presumably random. The epithelial cells of the exumbrella were then observed using DIC microscopy. Two representative examples of epithelial wound closure are shown (Fig. 2a-f, Additional files 1 and 2).

### Healing of epithelial wounds in *Clytia* is rapid and independent of cell proliferation

Wounds in *Clytia* closed extremely rapidly. We focused on wounds of 50–150 mm^2^. For wounds in this size range, the area was reduced by 50% in 4–8 min (Fig. 2g). The rate of closure, measured in wounds where the entire periphery could be seen (i.e. Fig. 2d-f), ranged from 50 to 118 μm^2^/s (~3–6 mm^2^/min; *n* = 6) based on the slope of a line approximating the change in wound area over time for the fastest and slowest healing wounds (Fig. 2h). By comparison, rates of 9–38 μm^2^/min are reported for *Drosophila* embryonic wound healing [10, 27], while MDCK cells and corneal epithelial monolayers in culture close a gap at a velocity of ~0.3 μm^2^/min ([6, 20, 28, 29]). Hence, the rate of wound closure in *Clytia* is ~75 to >600 times faster than rates reported for wound healing in embryos, and ≥10,000 time faster than in cultured cells.

The fast healing rate of *Clytia* epithelial wounds would seem to preclude the contribution of cell proliferation. Indeed, in intact animals, 5-ethynyl-2′-deoxyuridine (EdU) labeling demonstrated that division rates in the exumbrella epithelial cells of 2–3 week old animals are very low (Additional file 3), and no increase in EdU labeling was ever seen at wound sites (not shown). Furthermore, hydroxyurea treatment at levels that completely inhibited cell division had no effect on wound healing (Additional files 4 and 5). The lack of involvement of cell proliferation in gap closure is consistent with in vitro and in vivo findings in other systems that small wounds close without any cell proliferation, while in large wounds proliferation is stimulated only after closure of the gap [30–32].

### Healing of epithelial wounds by lamellipodia-dependent cell crawling is a three-phase process

**Fig. 3:**
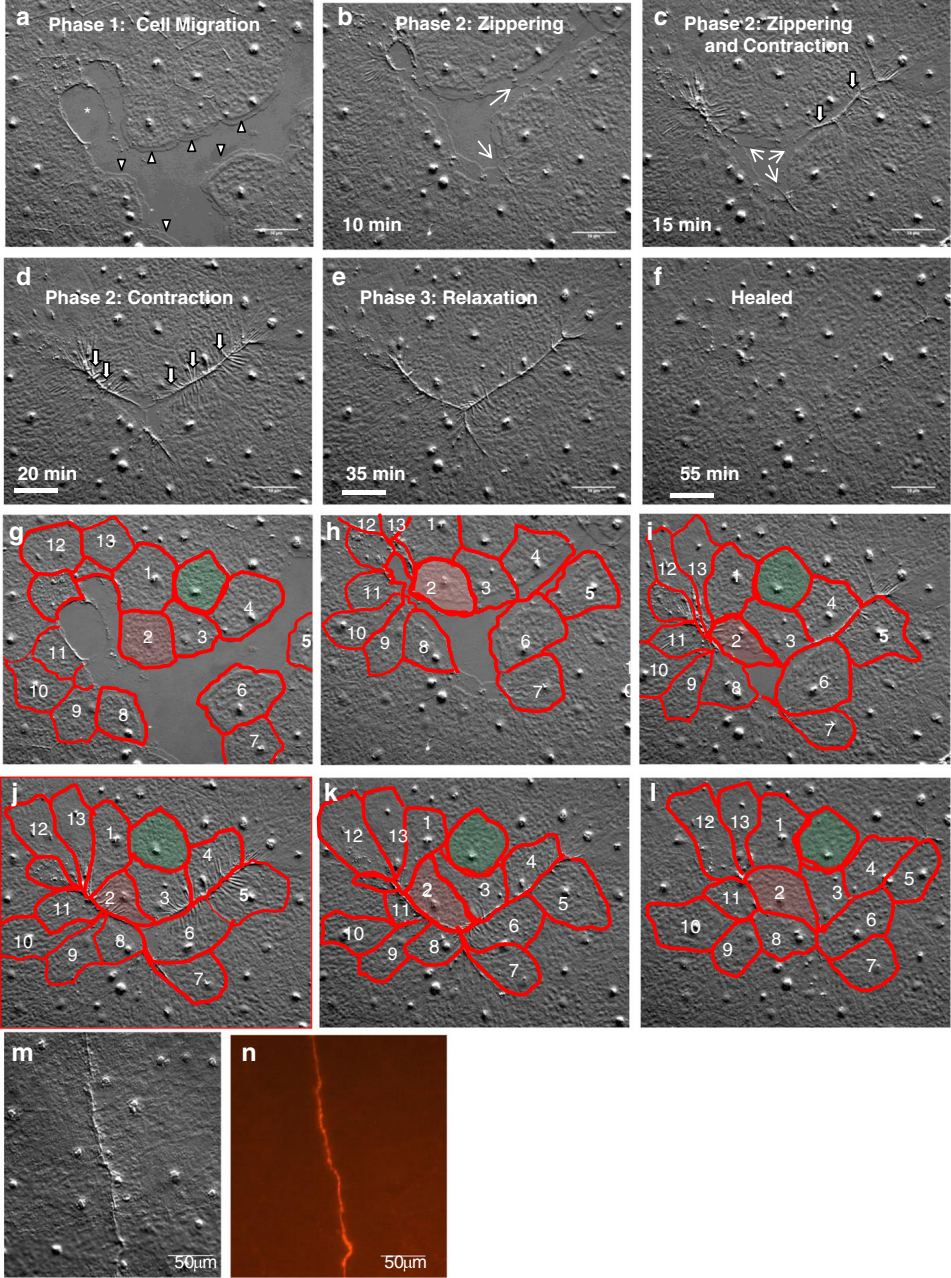
Epithelial wound healing by lamellipodia-dependent cell migration has three distinct phases. **a** Lamellipodia are clearly evident in Phase 1 (arrowheads). (Note that in the upper left portion of the wound there is a tear in the basement membrane (*) which heals by a different mechanism – see text). **b** In Phase 2, lamellipodia begin to meet across the wound gap. The region of contact is extending by “zippering” (arrows). **c** Puckers in the area that “zippered” indicate that contraction has begun (arrow indicate zippers, outlined arrows indicate contractions). **d** Contractions continue along the length of the closed wound (outlined arrows). **e** Reduced “puckering” indicates the progressive relaxation of contractions. Areas that contracted first also relaxed first. **f** At 55 min, the epithelial sheet is intact and no scarring is apparent. **g**-**l** Tracing of the perimeters of the cells shown in **a**-**f**. Wound marginal cells change shape (e.g. cell 2, highlighted in red) and position (e.g. cells 8–11) dramatically during wound healing. Much of the shape change is associated with contraction of the edge of the cell at the wound periphery. The shape change persists even after the wound is healed. Interestingly, cells that were not directly in contact with the wound (e.g. cell highlighted in green) show little if any shape change. **m**-**n** Actin accumulates at the site of a recently closed epithelial wound. **m** DIC of fixed cells after closure of a linear wound. The seam is a region where lamellipodia have zippered together. **n** Phalloidin staining of the cells in (**n**) shows accumulation of actin at the seam. Experiment was replicated >10 times. Scale bar = 50 μm

For the majority of epithelial wounds we observed, there was rapid formation of large projections in marginal cells (Figs. 2 and 3, Additional files 1 and 2). The appearance of these projections in DIC microscopy suggests that they are lamellipodia, and actin staining shows the expected network of actin fibers in these structures (Additional file 6). Careful observation of time lapse movies revealed that the healing process could be divided into three distinct and consistent phases: Phase 1) Lamellipodia formation, cell crawling and contact between the lamellipodia; Phase 2) “Zippering” between adjacent lamellipodia and contraction at the zipper; and Phase 3) relaxation of the wounded tissues and the re-establishment of an intact cell layer (Additional file 2).

#### Phase 1

**Fig. 4:**
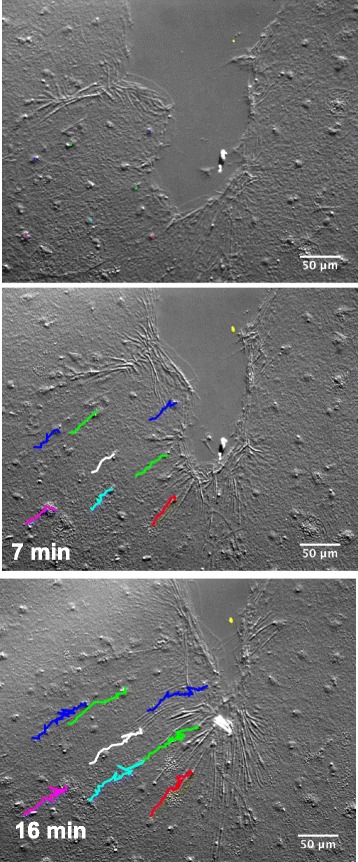
Collective cell migration occurs in a healing wound. Nuclei of individual cells were traced manually over a ~15 min time course. An imperfection in the mesoglea (yellow dot) creates a fixed position in the images and indicates that the sample was not drifting on the slide. Experiment was replicated 3 times. Scale bar = 50 μm

Phase 1 began with appearance of broad, ruffling lamellipodia emanating from marginal cells (Fig. 3a). Lamellipodia protruded 5–28 μm in front of the cell body (*n* = 100). Marginal cells migrated into the wounded area; nuclear tracking revealed that cells several tiers behind these cells also migrated (Fig. 4 and Additional file 7). The movement of the marginal cells and the cells behind them was smooth and coordinated with no jostling or rearrangement, characteristic of the sliding of cell sheets in collective cell migration [33]. Lamellipodia of marginal cells eventually met across the gap created by the wound. At this point, collective cell migration ceased.

**Fig. 5:**
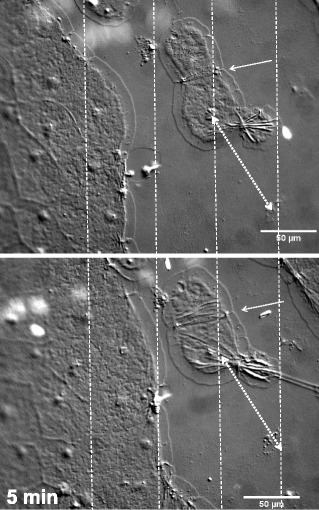
Isolated cluster of cells form lamellipodia but do not crawl. The 2 detached cells pictured compacted by zippering at their interacting edges (arrow). However they did not migrate, as indicated by the constant distance between the nucleus of the lower cell and an imperfection in the mesoglea (double arrow) after 5 min. Vertical gridlines help illustrate that the cells to the left have advanced towards the non-motile cell cluster in that time period. Experiment was replicated 3 times. Scale bar = 50 μm

Isolated cells or cell clusters occasionally became disconnected from epithelial cell sheets during wounding. These cells formed lamellipodia on all sides (Fig. 5, Additional file 8). However, these isolated cells never migrated, although cell pairs and small groups did “zipper” together (Fig. 5 arrows, see below) to maximize contact with each other along their edges (Additional file 8). It is therefore unlikely that these cells actively disconnected from the cell sheet. In contrast, our observations indicate that while the wound-induced signal for lamellipodia formation is effective in small groups of cells, this alone is not sufficient for directional migration. We conclude that either a larger number of contiguous cells or asymmetry in lamellipodia formation must be required for marginal cells to crawl.

#### Phase 2

*Zippering*. Migrating cells eventually met each other across the wound gap, but they often did not make contact with other cells along their entire length, nor did all cells on one side of a gap reach the cells on the other side synchronously. In most cases, portions of lamellipodia made contact with each other and the region of contact extended in a progressive zipper-like fashion (Fig. 3b, c and Additional file 2).

*Contraction.* Almost immediately after cells zippered together, lamellipodia disappeared and the newly formed seams contracted dramatically (Fig. 3c, d, Additional file 2). The axis of the contraction in linear wounds was always parallel to the length of the wound. The contractions extended across multiple marginal cells (Fig. 3c, d). However, contraction was not synchronous along the entire length of a wound, but occurred progressively as cells zippered.

Contraction was accompanied by dramatic changes in cell shape and position. Cells narrowed or became pointed at the closing wound edge, and some cells were jostled in their position relative to their neighbors (e.g. cells 2 and 10, Fig. 3g-l). Importantly, no contraction was ever seen in the tiers of cells behind the marginal cells, and there was little if any change in the shape of these cells (e.g. green cell in Fig. 3g-l).

Contractions in the context of wound healing usually are associated with super-cellular actin cables. To test whether the contraction phase of lamellipodia-mediated wound healing seen here is also associated with an actin accumulation, we fixed tissues after wounding and stained with phalloidin. We inevitably saw intense fluorescence at the site where wounds were in the process of healing (Fig. 3m,n). This is highly reminiscent of the actin accumulation seen during purse-string closure, and suggests that actin drives the contraction step.

#### Phase 3:

In Phase 3, an obvious relaxation of the wounded tissue revealed a newly intact epithelial sheet (Fig. 3e,f and Additional file 2). In longer linear wounds, the contraction and relaxation along the length of the wound was not synchronized but occurred progressively, with zippering, contraction and relaxation events moving as a wave down the length of the wound as it healed (Additional file 2). After relaxation, cells with a teardrop or wedge shape often revealed the previous existence of a wound (e.g. Fig. 3 f,l), but no scars were apparent.

### Contraction in Phase 2 requires non-muscle myosin II

**Fig. 6:**
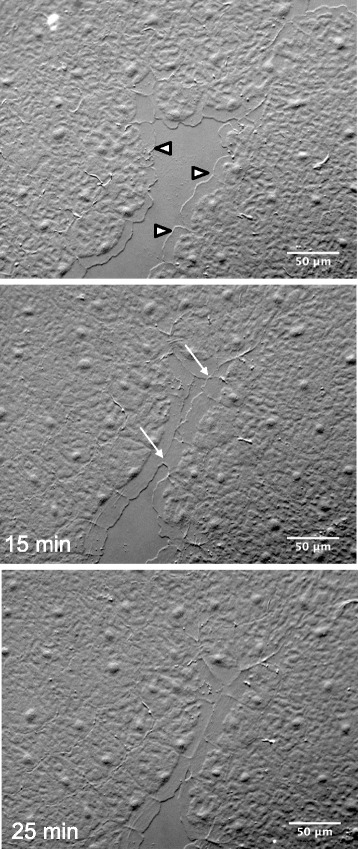
Non-muscle myosin II is essential for Phase 2 contraction. In the presence of blebbistatin, lamellipodia form (arrowheads) and close the wound gap, and zippering occurs between cells to create a seam (arrows). However, no apparent contraction follows the zippering even 10 min later (see Additional file 9 for greater detail). Experiment was replicated 3 times. Scale bar = 50 μm

The accumulation of actin in healing wounds suggests that an actomyosin-based mechanism may drive the Phase 2 contraction that follows lamellipodia zippering. To test the involvement of non-muscle myosin II, we added the inhibitor blebbistatin. Blebbistatin did not affect the formation of lamellipodia in marginal cells (Fig. 6, Additional files 9 and 10). Cells crawled and the lamellipodia met across the gap, as observed in untreated controls. However, there was no contraction following zippering, and no associated deformation of cell shape and size (Fig. 6, Additional files 9 and 10). In some cases wounds were still able to heal, while in other cases all healing ceased between zippering and contraction in Phase 2. Myosin II-dependent actin contraction has been described many times as part of purse-string wound closure [11, 34, 35]. However, here we found that myosin II-dependent actin cable contraction is associated with a non-purse string mechanism of healing.

### A classical purse string mechanism can also close epithelial wounds in *Clytia*

**Fig. 7:**
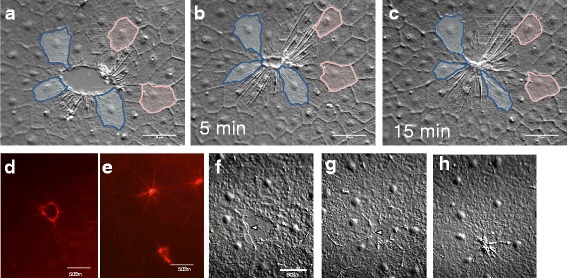
Wounds can close using a classic purse string mechanism. **a**-**c** Time lapse images of a wound closing without lamellipodia. There are small moving projections at the wound periphery, as well as clear contractions in marginal cells that precede gap closure. Marginal cells (blue) undergo major shape changes, while cells one to two tiers back (pink) do not (see Additional file 11 for timelapse movie). **d**,**e** Phalloidin staining of closing (**d**) and recently closed (**e**) wounds similar to those shown in **a**-**c** reveals actin accumulation at the circumference. **f**-**h** Wound of similar size and shape to (**a**-**c**) closing with lamellipodia (e.g. indicated by arrowheads). Contractions are only present after gap closure, as in the wounds in Figs. 2 and 3. See Additional file 12 for timelapse movie. Experiment was replicated >10 times. Scale bar = 50 μm

While the lamellipodia-mediated sequence of events described above was by far the most common wound closure mechanism that we observed in the *Clytia* epithelium, we sometimes saw circular wounds closing in the apparent absence of large, broad lamellipodia. Instead, small finger-like projections rapidly appeared and disappeared at the wound margins (Fig. 7a and Additional file 11) and actin accumulation was apparent around the circumference (Fig. 7d,e). In these wounds, contraction appeared to reduce the area of the wound gap long before the small projections were seen to contact each other, and there was no observable zippering. This sequence of events is identical to that described for purse string closure during embryonic wound healing, embryonic tissue remodeling and in some adult and in vitro contexts [10, 15, 18, 20, 36]. Marginal cells changed in shape during purse string closure as they were pulled forward and together to close the wound (e.g. blue cells, Fig. 7a-c). This typically created a “rosette” of wedge shaped cells to mark where a wound had been (Fig. 7c,e), as previously described ([20] and references therein). No shape changes were seen in cells located behind the marginal cells (e.g. pink cells, Fig. 7a-c).

The choice of a purse string mechanism to close a wound has been correlated with small circular shaped gaps [20, 21]. However, we found examples of both purse string and cell crawling mechanisms in wounds of similar shape and size (Fig. 7f-h, Additional file 12), suggesting that another factor must be important. Notably, even when small circular wounds closed by lamellipodia-dependent cell crawling, contraction at the seams was the final step (as with larger, linear wounds such as those in Fig. 3).

### Lamellipodia-dependent cell crawling requires an intact basement membrane

**Fig. 8:**
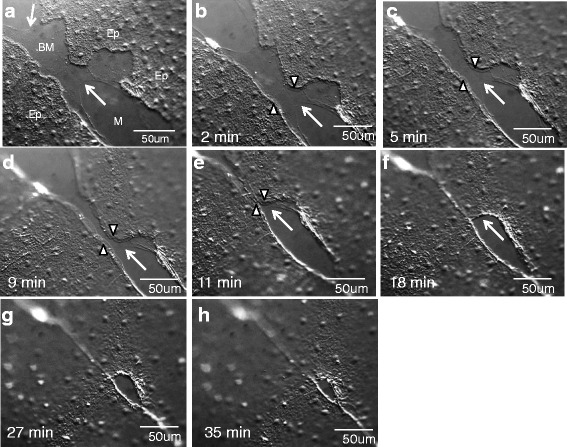
Tears in the basement membrane cause an abrupt shift from lamellipodia-dependent cell crawling to purse string mediated wound closure. **a**-**h** Wound healing in an intact animal with a torn basement membrane (BM). Arrows indicate the region where the basement membrane has torn, exposing the mesoglea (M)(**a**-**f**). Lamellipodia (arrowheads) are apparent as epithelial cells (Ep) move across the BM (**a**-**e**), but disappear when cells reach the torn area (**f**). The remaining wound is closed by a classic purse string mechanism (**g**, **h**). Experiment was replicated >10 times. Scale bar = 50 μm

If the presence or absence of lamellipodia-dependent cell crawling in marginal cells does not depend on wound shape or size in *Clytia*, what does it reflect? Interestingly, we occasionally observed wounds in which there was an abrupt shift from lamellipodia-dependent cell crawling to purse string closure. We reasoned that such wounds might provide clues to the factors that determine the wound healing mechanism. In cases where there was a shift in the closure mechanism, we noticed that lamellipodia disappeared when cells reached a region where the surface of the mesoglea (jelly) appeared disrupted (Figs. 8a and 3a (marked with *) and Additional file 13 show examples of such disruptions,). The uppermost layer of the ECM, basal to the epithelial cells, is the basement membrane (Fig. 1b,d). An example of a wound that involves a region of basement membrane damage is shown in Fig. 8 and in Additional file 13. Where the basement membrane was intact, epithelial cells formed lamellipodia (arrowheads) and crawled towards each other to close the gap (Fig. 8a-e, Additional file 13). However, cells did not crawl over the region that was denuded of a basement membrane. Instead, when cells reached the edge of the basement membrane tear, lamellipodia disappeared and cell crawling abruptly stopped (Fig. 8e-f and Additional file 13; absence of cells in the region lacking the basement membrane is apparent). Hence, it appears that interactions between the epithelial cells and the basement membrane are essential for cell crawling and maintenance of lamellipodia.

Almost immediately after cell crawling stopped, small finger-like projections appeared at the periphery of the tear, and the wound area reduced by contraction of the periphery in an apparent purse string mechanism (Fig. 8f-h, Additional file 13). This abrupt switch between wound healing mechanisms can also be seen in the closure of the wound in Fig. 3 and Additional file 2, in which a small basement membrane tear is apparent at the upper left (marked with an *). The region lacking basement membrane is surrounded by crawling cells that do not advance over the denuded region, and is then closed via a purse string. These finding indicate that the epithelial cells can rapidly convert between the two healing mechanisms, that lamellipodia formation is preferentially seen in the presence of a basement membrane, and that purse string closure does not require the presence of basement membrane in the wound gap.

## Discussion

This paper presents a detailed characterization of epithelial wound healing in a cnidarian species. Cnidarians, ctenophores (comb jellies) and porifera (sponges) are the earliest extant phyla to branch from the bilaterian lineage, and animals in each phylum have now been shown to have an epithelium and ECM, while cnidarians and many ctenophores also have a basement membrane [37–39]. It is therefore reasonable to expect that epithelial repair mechanisms are ancient, and that certain successful mechanisms evolved in a common ancestor and have been conserved in higher animals. Indeed, we observed many processes also seen in vertebrate wound healing, including collective cell migration, actinomyosin purse-strings, lamellipodia-dependent cell crawling and zippering of cells at the point of lamellipodia contact in the cnidarian *Clytia*. This supports the idea of ancient origin of these mechanisms. DuBuc et al. [40] showed that the ERK signaling pathway, known to be involved in wound healing in  *Drosophilia* and vertebrate cell culture, is involved in wound healing in the cnidarian *Nematostella*. This, together with the high level of conservation observed between cnidarians and vertebrates at the genetic level [41] suggests that wound healng in *Clytia* may be both morphologically similar to higher animals, and regulated by similar molecular pathways.

Despite similarities with wound healing in other animals, our studies in *Clytia* revealed some significant novel features. The most striking is speed; lamellipodia and purse strings form within minutes and wounds heal at a rate that is ~75 to ≥600 times faster than embryonic wound healing and ≥10,000 times faster than healing of artificial wounds in vitro. At this point we do not understand how *Clytia* heals wound at such an unprecedented rate, and this will be an important area for future research.

### Lamellipodia formation and not actin cable contraction distinguishes cell crawling from purse string-mediated wound healing

In this first characterization of *Clytia* wound healing, we report a contraction event in the healing of all observed epithelial wounds, even when closure is primarily driven by lamellipodia-dependent cell crawling. This is the first example we provide in this paper of a rapid switch of actin-based structures in a cell from lamellipodia to a contractile actin cable. Importantly, the role of actin cable contraction appears to be different in the two types of wound healing: in the purse-string mechanism, contraction of the actin cable pulls cells inwards to close the wound gap, while with cell crawling, the actin cable contraction only occurs after the wound has been closed by the zippering of lamellipodia. Notably, the actin cable in the latter case does not involve a circular wound or area of concavity, unlike purse strings previously described [15, 21, 32]. It would be interesting to determine where such a cable is anchored to allow it to generate the force needed for the contraction.

While the literature in general considers purse string and cell crawling mechanisms to be alternative wound healing mechanisms, [1] emphasized the idea that the reality is more complex, and summarized all descriptions in the literature of wounds that closed by a combination of purse strings and cell crawling ([1]). Our demonstration that actin cable contraction can play a role after the wound gap is closed is unique, to our knowledge, and adds yet another layer of complexity to this picture.

### Healing by lamellipodia-dependent cell crawling requires interactions between the epithelial cells and the basement membrane

Upon recognition of a wound, marginal epithelial cells make a choice between lamellipodia-dependent cell crawling and purse string mechanisms of healing. What might trigger such a choice?

Cell crawling requires interactions between cytoskeletal actin and proteins in the underlying substrate. In vivo, epithelial cells rest on a basement membrane, a distinct part of the ECM consisting primarily of a dense assembly of laminin and collagen IV [26]. Epithelial cell actin associates with integrins through various adaptor proteins, and integrins bind to ECM components such as laminin and fibronectin. The importance of ECM properties in cell migration has been extensively studied, and it is clear that both physical properties (i.e. stiffness) and molecular composition (i.e. growth factors, binding sites for integrins) are important (reviewed in [2]). The integrity of the basement membrane has been less considered. Our findings show that when the lamellipodia of crawling marginal cells encounter damage to the basement membrane, cell crawling abruptly stops and the lamellipodia rapidly disappear and are replaced by small finger-like projections and a purse string that circumscribes the wound. Hence, in *Clytia,* epithelial cell behavior is very different depending on whether or not the basement membrane is intact. It seems likely that the lack of interaction between cytoskeletal actin and basement membrane component(s) leads to the loss of lamellipodia, and we suggest that this triggers the “back up” purse string mechanism. In agreement with these ideas, [42] showed that in intestinal epithelial monolayers, co-culture with myofibroblasts that allowed the formation of a basement membrane contributed positively to wound healing. Furthermore, [23] showed that, in bovine corneal endothelial cells, the purse string vs. cell crawling choice depended on whether their wounding protocol removed (purse string) or retained (crawling) the basement membrane-like ECM secreted by these cells. Vedula et al. [18] further demonstrated in HaCaT cell monolayers that gaps devoid of all ECM proteins close exclusively by purse string, with a cell crawling-to-purse string switch observed at the edge of the gap. Together, these data suggest that the choice of wound healing mechanism is dependent on interactions between the cell and external cues such as basement membrane attachment. Interestingly, in corneal epithelial wounds studies in vivo, where the basement membrane was intact, disruption of actin cables with an antibody directed against a cadherin lead to a switch to lamellipodia formation [11]. This reinforces the idea that rapid mechanistic switches can occur in response to changes in external cues.

Interestingly, this is the second example in this paper of a rapid switch between actin-based structures. The existence of two wound healing mechanisms, and the ability to change rapidly between them, may provide robustness to the system and allow animals to cope with various types of epithelial damage.

### Future potential for the Clytia system as a wound healing model

Cnidarians have long been studied for their rapid healing and regenerative abilities ([25, 40, 43–45]). Furthermore, there is remarkable conservation of structural and regulatory genes between cnidaria and vertebrates [41]; this suggests that, despite their early divergence, studies in these animals will prove relevant to vertebrate systems. The small hydrozoan *Clytia hemisphaerica* has been recently adopted as a model for development and molecular evolution, and simple protocols for maintaining the animal in the lab are available [22]. Most importantly, its single layer of large squamous epithelial cells and its transparency allow easy, high resolution imaging in vivo.

Our results show that the basic events in the healing of epithelial wounds appear to be of ancient origin. It will next be important to determine if the molecular mechanisms underlying cell crawling and purse string closure in mammals are shared by *Clytia*. The potential of the system is currently limited by the lack of a protocol for making transgenic animals, an essential step in labeling the various relevant components so they can be watched during healing. However, CRISPR/Cas 9 technologies have already allowed genome manipulation in other Cnidaria such as Nematostella and Hydra [46, 47] and targeted mutagenesis has been achieved in *Clytia* [48]. Mutagenesis in particularly attractive in *Clytia* because it has a simple life cycle, and can be mated by mixing male and female medusae; hence, with the availability of mutants, classical genetic analyses could be used to probe signaling pathways involved in wound healing.

## Conclusions

*Clytia* offers a new tool to study the evolutionary origin of epithelial wound healing. The system allows analysis of wound healing processes common to all animals at high resolution without removing epithelial cells from their natural context in a live animal. The present study provides evidence that interaction between the basement membrane and epithelial cells is essential for cell crawling and maintenance of lamellipodia. In the absence of these critical interactions, as in the case of basement membrane damage, epithelial cells can rapidly switch to a purse-string mechanism of wound healing.

## Methods

### Animal culture

*Clytia* polyps were initially obtained as a gift from Tsuyoshi Momose and Evelyn Houliston, Observatoire Oceanologique de Villefranche sur Mer, and from Centre National de Ressources Biologiques Marines, EMBRC-France (http://www.embrc-france.fr). These polyps were used to establish colonies at the University of Chicago, and baby medusae were collected from these colonies as needed. *Clytia* were maintained in sea water at 18 °C in a Z-Hab mini system (Pentair) with 10 l zebrafish tanks for polyp colonies and custom-made 5 l kreisel tanks for medusae (contact authors for plans). Sea water (Red Sea Salt) was made at a concentration of 4% red sea salts with specific gravity adjusted to 1.027. 2–3 week old medusae were used for all experiments.

### Wounding of medusae

Animals were placed on a depression slide in sea water with the exumbrella facing upwards, towards the microscope lens. A pulled Pasteur pipette was used to gently abrade the surface of the animal. Animals were then relaxed in 0.1% Tricaine (Ethyl 3-aminobenzoate methanesulfonate, Sigma) made as a 1:10 dilution in sea water of the following stock: 200 mg Tricaine, 20 ml ddH2O, pH to 7.5 with NaOH. A cover slip was then placed on top. For blebbistatin treatment, blebbistatin stock (50 μM in DMSO) was diluted 1:10,000 in sea water and animals were incubated for 2 h before wounding. (Note that DMSO inhibits wound healing at higher concentrations). Blebbistatin was added at the same concentration to the tricaine used for mounting.

### Imaging of wound healing

Wound healing was imaged using a Leica HC microscope with standard DIC optics. Pictures were taken at 12 s intervals. Timelapse movies were created using moviemaker (http://windows-movie-maker.org/). Wound areas were measured using FIJI (http://imagej.net/Fiji/Downloads). For rate of closure, the wound area was measured in frames 2 min apart for 6 wounds and plotted and the slope was calculated using the “trendline” function of Microsoft Excel.

### Electron microscopy

Newly released medusae were transferred to aluminum planchettes (Ted Pella) containing sea water, cryo-fixed in a High Pressure Freezer (HPM 010;RMC), and placed immediately into cryo-tubes containing a frozen cocktail of 0.2% uranyl acetate in anhydrous acetone. All frozen samples were placed into an Automatic Freeze Substitution Machine (AFS2, Leica), and freeze substituted at −80 °C for 36, warmed to and held at −50 °C for all remaining processing steps. The samples were washed three times with acetone and then slowly infiltrated with Lowicryl HM20 mono-step resin [Electron Microscopy Sciences (EMS)] according to the following schedule: 25, 50, 75, and 100% (8 h at each concentration) followed by three 1-h 100% resin washes. The samples were placed into flat-bottomed molds (EMS) and polymerized at −50 °C under UV light for 20 h.

Samples were remounted so that cross-sections through the animal would be obtained. Samples were cut into multiple 80-nm sections using an ultramicrotome (UC6, Leica) and mounted on Formvar-carbon coated copper slot grids (EMS). Images were collected using a transmission electron microscope operated at 120 kV (Tecnai Spirit; FEI).

### Measurement of cell division rates

Click-iT EdU Alexa Fluor 555 Imaging Kit (ThermoFisher) was used to assess cell division rates. Animals were labelled with EdU for 24 h with or without hydroxyurea at a final concentration of 20 mM.

### Actin staining

Phalloidin was used to image actin. Animals were relaxed with Tricaine, fixed in formaldehyde for 10 min (250 μl 37% formaldehyde, 1 ml sea water, 9 ml water), washed 2× in sea water, permeabilized 10 min in 0.1% Triton X-100 in sea water, and washed an additional two times in sea water. They were then stained in 2.5% plalloidin (Alexa Flour 555 Phalloidin, Thermo Fisher) in sea water for 1 h-overnight, washed in sea water and imaged.

## Additional files

Additional file 1: Movie 1. Wound healing time lapse of the wound pictured in Fig. 2a–c. Frames were taken every 16 s. The duration of the movie is 20 min. Scale bar = 50 μm. (MP4 21,097 kb)

Additional file 2: Movie 2. Wound healing time lapse of the wound pictured in Fig. 3a–f. The major events during each of the phases, as described in the text, are labeled. Frames were taken every 12–13 s. The duration of the movie is 55 min. Scale bar = 50 μm. (MP4 27,840 kb)

Additional file 3: Figure S1. Labeling index of cells in the exumbrella. EdU incorporation showed that in the first 3–4 days after release there is very little, if any, division in epithelial cells in the exumbrella. In contrast, at 7 days the percentage of cells dividing within a 24 h period is >40%. By two and three weeks, the number of dividing cells per 24 h is greatly reduced, and declines further as animals age. Therefore, in the 2–3 week old animals used in wounding assays there is little epithelial cell division in the exumbrella. Animals were labelled for 24 h with EdU, and then fixed and stained with Hoescht stain. Values are the percentage of Hoechst stained cells that also showed EdU labeling. 3–5 animals were examined at each time point. Error bars = s.e.m. (PPTX 53 kb)

Additional file 4: Figure S2. 20 mM hydroxyurea treatment completely inhibits cell division in the Clytia medusa exumbrella. 7 day old animals were labeled with EdU for 24 h in the absence (A,B) or presence (C,D) of 20 mM hydroxyurea. B and D show Hoechst staining in the same animals in A and C, respectively. Scale bar = 50 μm (PPTX 824 kb)

Additional file 5: Movie S1. Wound healing time-lapse in the presence of hydroxyurea at concentrations shown to completely inhibit cell division (Additional file 4). Frames were taken every 12–13 s. The duration of the movie is 11 min. Scale bar = 50 μm. (MP4 11,528 kb)

Additional file 6: Figure S3. Actin in lamellipodia of epithelial cells at a wound site. Wounded animals were fixed and stained with phalloidin, and imaged using a Zeiss 710 laser confocal microscope. Lamellipodia can be seen extending from intact marginal cells and from pieces of cells in the wound gap. Scale bar = 50 μm. (PPTX 879 kb)

Additional file 7: Movie 3. Cell migration time-lapse of the wound pictured in Fig. 4. Nuclei were tracked manually using the Tracker function of FIJI. The yellow dot identifies an imperfection in the mesoglea that does not move, and therefore serves as a reference point for the movement of cells. Frames were taken every 12–13 s. The duration of the movie is 16 min. Scale bar = 50 μm. (MP4 14,138 kb)

Additional file 8: Movie 4. Wound closure time-lapse of the wound pictured in Fig. 5b. A small cluster of cells has become disconnected from the sheets on either side. Note that the lamellipodia of these two cells zipper, bringing the cells closer together. However, the isolated cells do not migrate, and are captured by the migrating sheets on either side. There is some drifting of the specimen, but a reference point of a mesoglea defect can be used to account for this, as shown in Fig. 5. Frames were taken every 12–13 s. The duration of the movie is 17 min. Scale bar = 50 μm. (MP4 8198 kb)

Additional file 9: Movie 5. Wound closure time lapse in the presence of 5 μM blebbistatin as pictured in Fig. 6. Cells migrate and lamellipodia form, meet and zipper. However, the characteristic contraction that follows zippering is absent. While occasionally treated wounds healed, the wound in this movie never heals. Frames were taken every 12–13 s. The duration of the movie is 25 min. Scale bar = 50 μm. (MP4 5861 kb)

Additional file 10: Movie S2. Wound healing time lapse in the presence of DMSO (1:10,000 dilution), the solvent used for blebbistatin (Fig. 6, Additional file 9). Frames were taken every 12–13 s. The duration of the movie is 15 min. Scale bar = 50 μm. (MP4 2907 kb)

Additional file 11: Movie 6. Purse string closure time-lapse of the wound pictured in Fig. 7A–C. Frames were taken every 12–13 s. The duration of the movie is 15 min. Scale bar = 50 μm. (MP4 822 kb)

Additional file 12: Movie 7. Lamellipodia-mediated closure of a wound of the same approximate size and shape as in Additional File 11, as shown in Fig. 7F–H. Note that after lamellipodia meet to close the gap, there is a contraction around the perimeter. When looking at a healing wound in this contracted state it is impossible to tell whether the wound originally closed through lamellipodia meeting (this movie) or a purse string drawing the cells forward (Additional file 11). Frames were taken every 12–13 s. The duration of the movie is 10 min. Scale bar = 50 μm. (MP4 5766 kb)

Additional file 13: Movie 8. Healing of an epithelial wound where there is a visible tear in the basement membrane (arrow heads). Lamellipodia can be seen to migrate over the area of the wound where the basement membrane is intact (right hand side), but only small finger-like projections are seen in the area that is denuded of the basement membrane. Once lamellipodia meet, the remainder of wound healing appears to occur primarily through a purse string closure mechanism. Frames were taken every 12–13 s. The duration of the movie is 32 min. Scale bar = 50 μm. (MP4 18,233 kb)

## Abbreviations

DIC: Differential Interference Contrast
ECM: Extracellular matrix
