# Supplementary figures and images for: In vivo imaging of epithelial wound healing in the cnidarian Clytia hemisphaerica demonstrates early evolution of purse string and cell crawling closure mechanisms
Source: BMC Dev Biol. 2017 Dec 19;17:17. doi: 10.1186/s12861-017-0160-2 (PMC5735930; doi:10.1186/s12861-017-0160-2)

## Slide 1
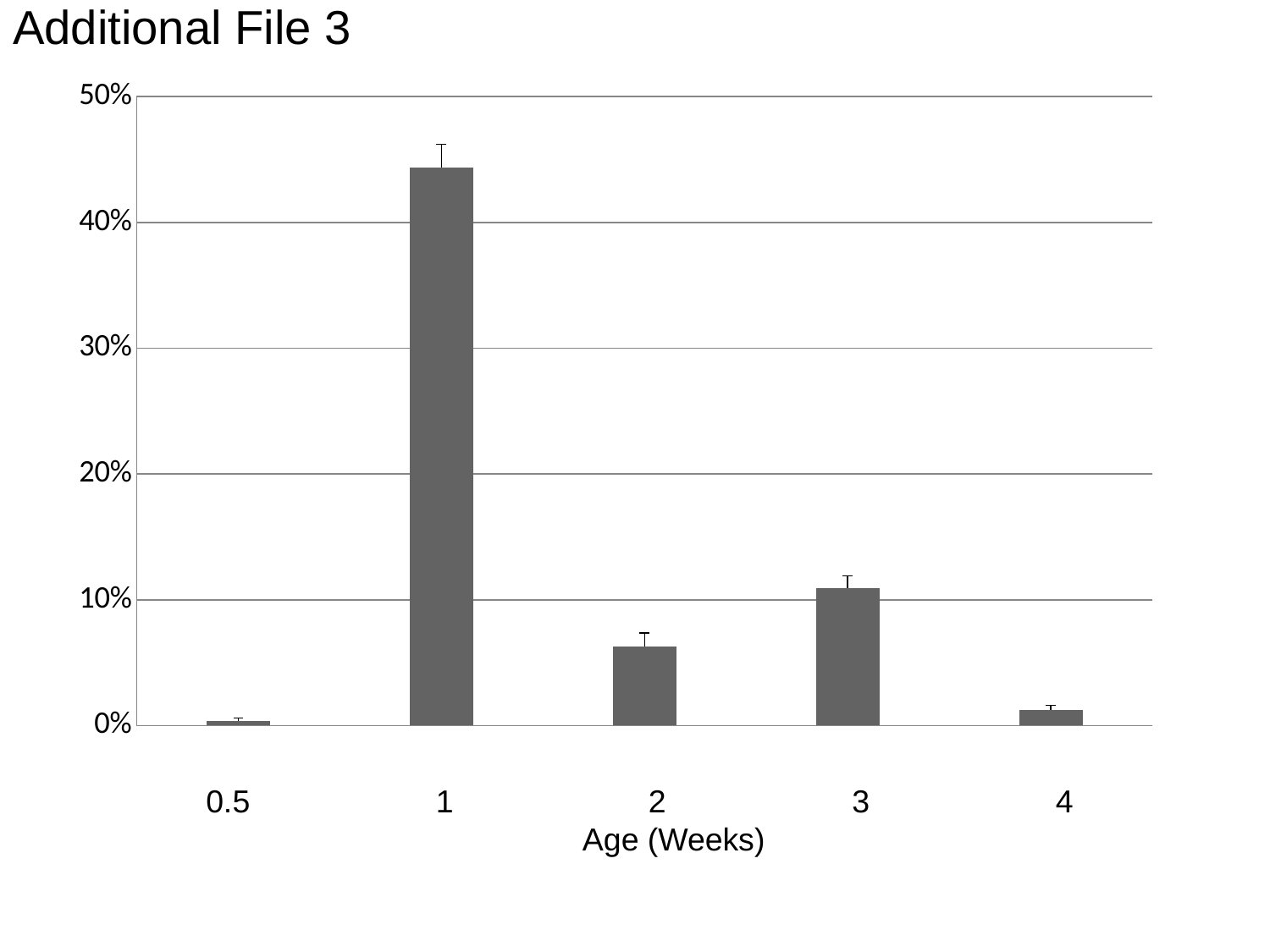

# Additional File 3
### Chart
| Category | mean (%) |
|---|---|0.5 1 2 3 4
Age (Weeks)

Supplement: Supplementary file 3 — Labeling index of cells in the exumbrella. EdU incorporation showed that in the first 3–4 days after release there is very little, if any, division in epithelial cells in the exumbrella. In contrast, at 7 days the percentage of cells dividing within a 24 h period is >40%. By two and three weeks, the number of dividing cells per 24 h is greatly reduced, and declines further as animals age. Therefore, in the 2–3 week old animals used in wounding assays there is little epithelial cell division in the exumbrella. Animals were labelled for 24 h with EdU, and then fixed and stained with Hoescht stain. Values are the percentage of Hoechst stained cells that also showed EdU labeling. 3–5 animals were examined at each time point. Error bars = s.e.m. (PPTX 53 kb) [file 12861_2017_160_MOESM3_ESM.pptx]

## Slide 1
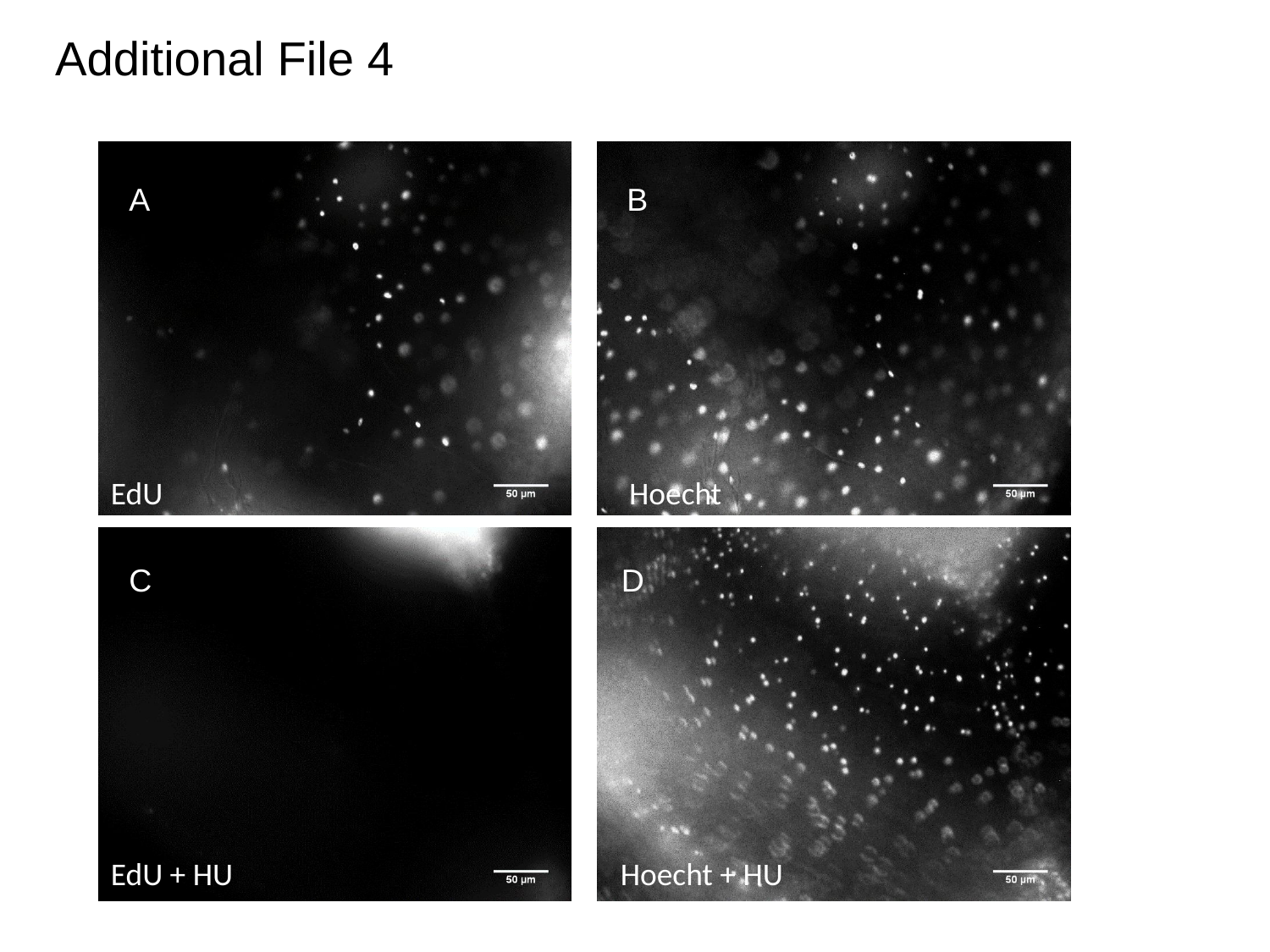

# Additional File 4
A B
C D
EdU Hoecht
EdU + HU Hoecht + HU

Supplement: Supplementary file 4 — 20 mM hydroxyurea treatment completely inhibits cell division in the Clytia medusa exumbrella. 7 day old animals were labeled with EdU for 24 h in the absence (A,B) or presence (C,D) of 20 mM hydroxyurea. B and D show Hoechst staining in the same animals in A and C, respectively. Scale bar = 50 μm (PPTX 824 kb) [file 12861_2017_160_MOESM4_ESM.pptx]

## Slide 1
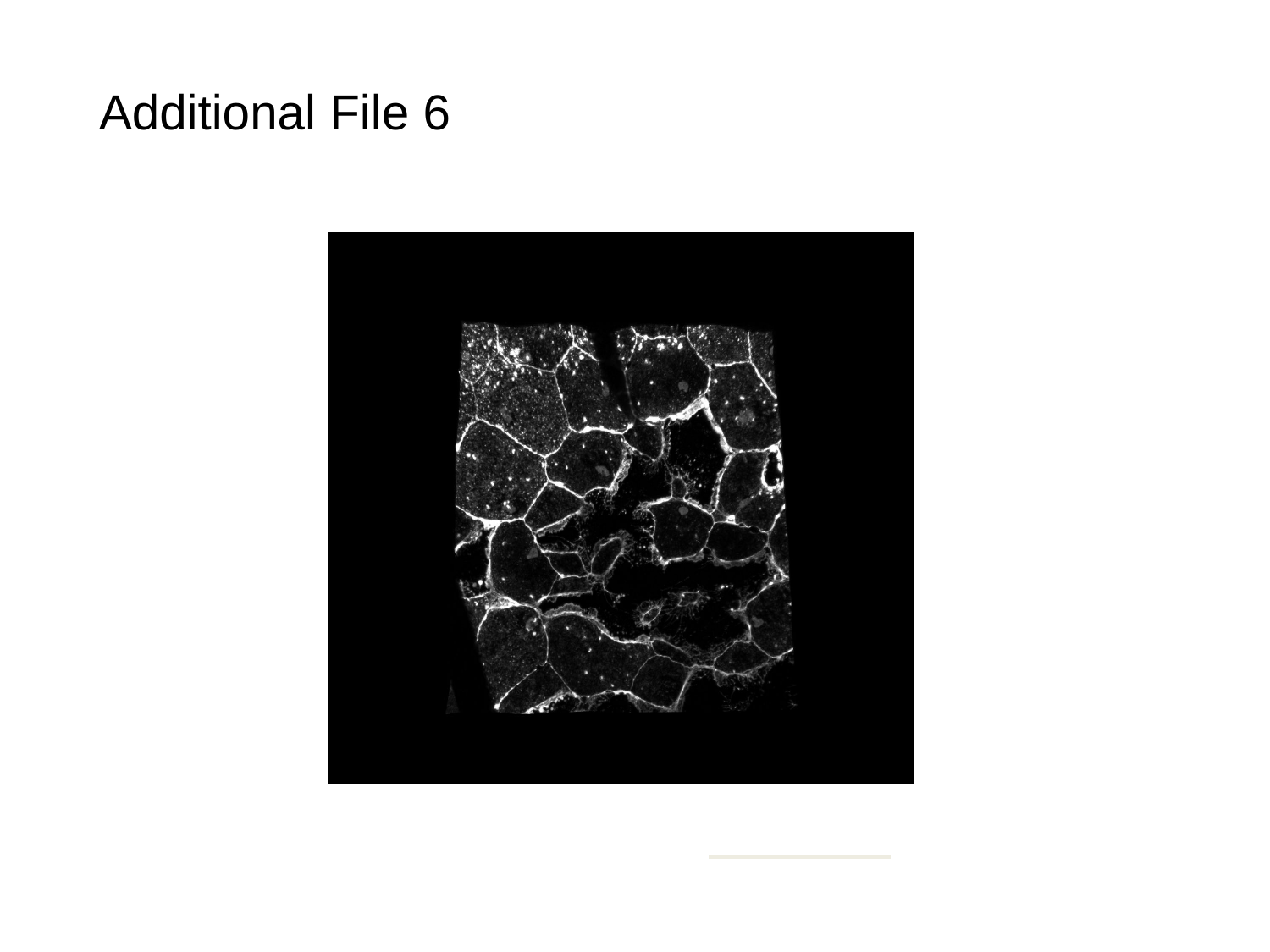

Additional File 6
50 mM

Supplement: Supplementary file 6 — Actin in lamellipodia of epithelial cells at a wound site. Wounded animals were fixed and stained with phalloidin, and imaged using a Zeiss 710 laser confocal microscope. Lamellipodia can be seen extending from intact marginal cells and from pieces of cells in the wound gap. Scale bar = 50 μm. (PPTX 879 kb) [file 12861_2017_160_MOESM6_ESM.pptx]
